# Infection prevention and control measures for emerging infectious disease: lessons learned from the first case of imported Lassa fever in China

**DOI:** 10.1186/s13756-025-01597-4

**Published:** 2025-07-01

**Authors:** Yalan Peng, Yantong Wang, Shiyu Li, Jing Huang, Wu Shi, Ruocheng Luo, Xianmou Pan, Wenzhi Huang, Fu Qiao, Yi Chen

**Affiliations:** 1 Center of Infectious Diseases, West China Hospital, Sichuan University, Chengdu, 610041 China; 2 Department of Infection Control, West China Hospital, Sichuan University, Chengdu, 610041 China; 3 Department of Infection Control, West China Xiamen Hospital of Sichuan University, Xiamen, 361000 China; 4 Department of Infection Control, West China Tianfu Hospital, Sichuan University, Chengdu, 610218 China

**Keywords:** Lassa fever, Infection prevention and control, Epidemiological investigation, Public health strategy

## Abstract

**Background:**

Lassa fever is an acute viral hemorrhagic disease prevalent in West Africa. West China Hospital of Sichuan University (WCHSCU) received China’s first imported case of Lassa fever from overseas. We described the epidemiological investigation and infection prevention and control measures of this case following the confirmed diagnosis.

**Methods:**

An emergency epidemiological team defined close contacts and implemented infection prevention and control (IPC) measures: (1) isolation in a designated isolation room, (2) environmental disinfection for high-, medium-, and low-risk areas, (3) 21-day quarantine for contacts. Data were collected via field observations and medical records.

**Results:**

We identified 6 close contacts and 74 general contacts, who were subsequently quarantined for 21 days. The hospital environment was classified into high, medium, and low-risk areas, and corresponding cleaning and disinfection measures were implemented. Ultimately, no new infection cases emerged.

**Conclusion:**

Rapid risk stratification, strict isolation protocols, and multidisciplinary coordination effectively prevented transmission, underscoring the importance of preparedness in non-endemic regions.

**Supplementary Information:**

The online version contains supplementary material available at 10.1186/s13756-025-01597-4.

## Introduction

### Global context and China’s first imported Lassa fever case

Lassa fever is a zoonotic disease, known to be endemic in Benin, Ghana, Guinea, Liberia, Mali, Sierra Leone and Nigeria. The latter scenario poses a risk of human-to-human transmission, particularly in hospitals and in laboratories [1]. Symptoms often develop gradually, beginning with fever, general weakness, and discomfort, and may progress over several days to include headache, sore throat, muscle pain, chest pain, nausea, vomiting, diarrhea, coughing, and abdominal pain. In advanced stages of the disease, patients may experience shock, seizures, disorientation, and coma. The mortality rate for Lassa fever ranges from 1–15% [1]. Patients with acute Lassa fever usually take 1 to 3 weeks to recover, however, even after recovery, they may experience sequelae of the disease, such as polyserositis, vision distortion, vertigo, hearing loss and back pain [2, 3].

Hospital-acquired infections caused by Lassa fever are not uncommon. The disease was first identified in 1969 when three missionary nurses contracted it at a Nigerian hospital, subsequently transmitting it to two colleagues during patient care, marking the earliest documented nosocomial outbreak. In 1997, Nigeria reported hospital-acquired infections of Lassa fever among healthcare workers due to the lack of gloves in hospitals [4–6]. Strengthening infection prevention and control measures effectively reduces the hospital-acquired infection rate of Lassa fever [7].

Since the discovery of the Lassa virus in 1969, China had not reported any cases of Lassa fever. However, in 2024, West China Hospital of Sichuan University received the country’s first case. This study documents the emergency infection prevention and control measures implemented, and subsequent adjustments made to public health strategies. The insights garnered from this case hold significant implications for healthcare facilities that may encounter Lassa fever in the future, particularly concerning strategies for infection prevention and control.

### Epidemiological history of the patient

**Fig. 1:**
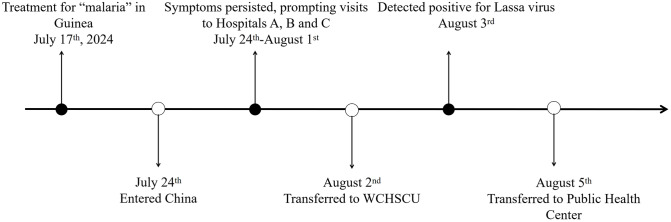
Diagram of case movement trajectory

The patient was a 49-year-old female who traveled to Guinea, Africa, in March 2024 for work. On July 17th, while still in Guinea, the patient presented with symptoms of fever and poor appetite. Upon visiting a local hospital, malaria parasites were detected, leading to diagnoses of both malaria and typhoid fever. The patient was treated with artemisinin and levofloxacin, resulting in an improvement in her condition. On July 23rd, she returned to China from Guinea. On July 24th, the patient sought care at Hospital A, reporting symptoms such as backache, abdominal pain, frequent and urgent urination, nausea, vomiting, and dry mouth. On July 25th, the patient was discharged after one night’s stay without receiving any medicine. Beginning on July 30, the patient exhibited symptoms, including sluggishness, slurred speech, incontinence, difficulty swallowing, and abdominal pain. Subsequently, the patient was transported via ambulance to Hospital B for treatment, she was diagnosed with sepsis and multiple organ dysfunction syndrome. On August 1, due to lack of improvement in symptoms despite treatment, she was transferred to Hospital C by ambulance. A blood smear test for malaria parasites returned negative results. Additionally, lumbar puncture and cerebrospinal fluid Next Generation Sequencing (NGS) tests were performed. Given the patient’s recent history of malaria in Africa, the potential for a malaria relapse could not be excluded. On August 2, due to recurrent fever and a continuous decline in platelet count, the patient was transferred to the emergency department of West China Hospital of Sichuan University for further treatment. From August 2 to August 3, the patient underwent blood tests for malaria parasite and malaria parasite antigen testing, both of which returned negative results. A lumbar puncture was also performed, and the cerebrospinal fluid culture submitted for testing returned negative as well. On the evening of August 3, the hospital received a notification from the local Center for Disease Control and Prevention (CDC) that the cerebrospinal fluid NGS test conducted during the patient’s treatment at another hospital indicated a positive result for Lassa virus. On August 5, the patient was transferred to the Public Health Clinical Center of Chengdu (PHCC) for isolation and treatment (Fig. 1).

## Method

### Epidemiological investigation

Following the notification from the CDC, the Infection Control Department immediately initiated an epidemiological investigation within the hospital. This task was undertaken by the hospital’s Emergency Epidemiological Investigation Team, which has been established since 2020 [8]. The investigation was conducted simultaneously in three areas. First, to identify the diagnostic and treatment procedures where infection transmission may have occurred. Second, to assess the risk of environmental contamination within the hospital. And third, to evaluate the risk of infection among hospital staff.

Specifically, the epidemiological investigation comprised four steps.

The first step involved a detailed listing of the patient’s medical treatment procedure during their hospital stay. This includes a comprehensive review of all electronic medical records, including progress notes, nursing records, procedure records, surgical records, and examination records. Additionally, on-site or telephone interviews with the attending physician and nurse responsible for the patient’s care was conducted to supplement the details found in the medical records. After gathering the information, the patient’s medical treatment procedure was organized chronologically, outlining the time points, locations, procedures performed, and types of personnel involved in the patient’s diagnosis and treatment.

The second step involved creating a list of potential exposed personnel and the environments where the patient stayed. Utilizing the patient’s treatment trajectory as a guide, compiled the list of staff members who had contact with the patient at each stage through phone interviews, reviewing shift schedules, and examining information system records. This list included shift workers, temporary interns, and other mobile personnel (e.g. waste collectors, itinerant professional caregivers). Additionally, verifying the environments where the patient stayed by interviewing staff who interacted with the patient.

The third step involves investigating the contacts between potential exposed personnel and the patient, as well as assessing whether the environments where the patient stayed were contaminated. Investigation methods include administering questionnaires to potentially exposed personnel and conducting phone interviews. Inquiries should focus on their contact with the patient’s blood, body fluids, secretions, or excretions, and whether personal protective equipment was used during such interactions. Additionally, assess the possibility of environment contamination by the patient’s blood, body fluids, secretions, or excretions during medical procedures. Some personnel may unintentionally omit important contact details during the initial investigation. Therefore, for those suspected of high-risk exposure, it is necessary to repeatedly confirm their contact status. In this epidemiological investigation, we conducted phone interviews with personnel who reported potential contact with the patient in the questionnaire. For those who had a higher likelihood of exposure to blood, body fluids, or secretions, we helped them recall potential exposure scenarios by listing specific interactions. For instance, we asked caregivers whether they brushed the patient’s teeth or emptied their urine, and we asked medical laboratory staff whether they performed handling and centrifugation of the patient’s samples in a biological safety cabinet. During the phone interviews, we discovered that several staff members who initially reported no contact with body fluids on the questionnaire were later found to have had actual contact.

The fourth step involves risk assessment and classification. The criteria for assessing the risk of exposure personnel are determined through consultation by the epidemiological investigation team, based on the transmission routes of Lassa fever and relevant literature on epidemiological investigations of the disease. We classify the risk levels of exposed personnel into three categories: close contacts, general contacts, and unexposed personnel [9, 10] (Table 1). All members of the investigation team collaborate to assign a risk rating to each exposed individual according to the established criteria.

**Table 1:** Level of risk for personnel resulting from exposure to a patient with Lassa fever

Risk Category	Description	Example
Close contacts	Unprotected exposure of skin or mucous membranes to potentially infectious blood or body fluids, or unprotected handling of clinical/laboratory specimens	λ Emptied the patient’s urine without proper protective measures. λ Collected or handled the patient’s blood or bodily fluid samples without proper protective measures.
General contacts	Close direct contact with the case, while using appropriate personal protective equipment(PPE), or not handle body fluids.	λ Measured the patient’s body temperature. λ Turned the patient over while wearing work clothes and gloves.
Unexposed personnel	No contact with the case	λ Worked in the patient’s ward without direct contact with the case. λ Performed a CT scan on the patient without direct contact with the patient.

### Infection prevention and control measures

#### Hospital infectious disease pre-screening and triage

The clinical departments in the hospital includes a fever clinic, a diarrhea clinic, and an infectious disease center. The fever clinic is equipped with a negative pressure emergency room and is capable of admitting patients with various infectious diseases. High-risk patients are triaged to either the fever clinic or the diarrhea clinic through temperature screening, epidemiological history, and symptom inquiry. Patients remain isolated and treated until potential risks are ruled out. The hospital’s specialized emergency consultations and multidisciplinary consultations ensure timely and effective treatment. Once the risks have been excluded, patients are transferred to general wards for ongoing care and treatment.

#### Routine precautions

Standard precautions and transmission-based precautions are implemented throughout the hospital. All clinical areas are required to be stocked with sufficient PPE to ensure easy access by healthcare workers. Regular training on the use of PPE and hand hygiene is provided for both existing staff and new employees. The infection control department conducts periodic checks to ensure staff compliance with these measures.

#### Cleaning and disinfection

The hospital has established specific cleaning and disinfection protocols, including frequency and method requirements for different areas. For surfaces in general wards, disinfection is carried out once a day. In high-risk departments or protective isolation areas, such as the fever clinic, emergency department, infectious disease center, and intensive care unit, disinfection is done twice a day. Additionally, infection control nurses from the department and the hospital’s infection control department regularly conduct fluorescent monitoring and sampling tests to check the effectiveness of cleaning and disinfection practices.

#### Vector control

The hospital implements rodent control routinely. Clinical waste is stored in sealed containers away from treatment areas and removed promptly, with daily disinfection of storage zones. Rodent-proof nets cover sewage outlets and entry points. Stagnant water and debris near facilities are routinely removed, and green areas adjacent to buildings are maintained. Real-time monitoring is conducted, and if the risk of disease transmission by a specific vector increase, disinfection in key areas is immediately intensified.

## Results

### Emergency response measures in hospital

#### Patient isolation

Upon receiving notification from the CDC that a cerebrospinal fluid NGS test from another facility indicated the presence of Lassa virus, the emergency department was immediately informed about the case. Due to limited availability of isolation rooms in the emergency department, which were already occupied by other patients requiring isolation, the hospital decided to transfer the case to a designated isolation room in the fever clinic. The fever clinic is managed by the emergency department, located adjacent to it, and is equipped with isolation rooms and a clearly defined workflow. Following patient isolation, a dedicated team comprising 3 physicians, 5 nurses, and 2 environmental services staff was deployed to manage care, minimizing personnel exposure. All team members completed infection control training prior to deployment, including N95 fit-testing. All personnel entering the patient’s room were required to wear medical protective masks (N95), gloves, medical caps, and medical disposable coveralls. In cases where large splashes of blood, body fluids, or secretions were possible, face shields or goggles were required. Strict zones were established for donning and doffing personal protective equipment (PPE), and movement pathways were carefully planned to avoid cross-contact with other personnel.

### Cleaning and disinfection

#### Emergency cleaning and disinfection

After the patient was transferred to the isolation ward, an emergency cleaning and disinfection process was immediately carried out. The scope of disinfection was determined based on the findings of the epidemiological investigation. According to the patient’s trajectory within the hospital and the risk of exposure to blood, body fluids, secretions, or excretions in each area, the appropriate level of cleaning and disinfection intensity was applied (Table 2). A dedicated cleaning and disinfection team was formed, comprising infection control professionals, cleaning supervisors, ward nurses, and cleaning staff. Infection control professionals were responsible for defining the scope of cleaning and disinfection, guiding the disinfection methods for each area, and evaluating the effectiveness of the disinfection. The cleaning supervisor organized the cleaning staff according to the workload to perform the emergency disinfection tasks. Ward nurses monitored the progress and effectiveness of the cleaning and disinfection within the ward, while the cleaning staff executed environmental disinfection duties. Environmental sampling targeting high-risk zones was performed using RT-PCR testing. A total of 15 samples were collected from: Emergency Resuscitation Bay 2&3, debridement Room and patient-used bathroom.

**Table 2:** Level of risk to the environment resulting from exposure to a patient with Lassa fever

Risk Category	Description	Example	Disinfection methods
High risk	Areas that may be contaminated by the blood, body fluids, or secretions of the case	λ The bed that the patient had previously used λ The debridement room where the lumbar puncture procedure was performed on the patient λ The toilet where the patient’s urine was emptied λ The laboratory where tests are conducted on the patient’s blood and body fluids	Clean and disinfect environmental surfaces and floors using 2000 mg/L chlorine-based disinfectant with a minimum contact time of 60 min.
Medium risk	The area where the patient has been, but has not been contaminated by blood, body fluids, or secretions	λ The CT room where the patient underwent examination	Clean and disinfect environmental surfaces and floors using 1000 mg/L chlorine-containing disinfectant with a minimum contact time of 60 min.
Low risk	The area where the patient has not been present but may have been indirectly contaminated	λ The beds of other patients in the same ward as the case	Clean and disinfect environmental surfaces and floors using 500 mg/L chlorine-containing disinfectant with a minimum contact time of 60 min.

#### Daily cleaning and disinfection of the isolation room during the patient’s isolation

During the patient’s isolation in the fever clinic, one designated cleaning staff member was assigned to clean and disinfect the room. Prior to commencing work in the isolation room, the cleaning staff member received training on PPE from infection control professionals. During their shift, the ward nurse was responsible for guiding the cleaning staff on the proper donning and doffing of PPE to avoid occupational exposure. The cleaning staff cleaned and disinfected the surfaces in the isolation room twice daily, using a 2000 mg/L chlorine disinfectant solution.

#### Terminal disinfection after patient discharge

After the patient was transferred, terminal disinfection of the isolation room in the fever clinic was performed using a hydrogen peroxide fogging machine. The effectiveness of the disinfection was evaluated using adenosine triphosphate (ATP) testing.

Infection Prevention and Control Measures for Patient Transfer and Isolation at PHCC.

The patient was transferred to PHCC via a negative-pressure ambulance, with the transport route planned by IPC personnel to avoid contact with others. Healthcare workers assisting the transfer wore full PPE including N95 respirators, protective gowns, caps, and gloves in accordance with WHO guidelines. Upon arrival, the patient was isolated in a negative-pressure room where all healthcare workers wore full PPE during contact. The designated care team was centrally accommodated at an isolation hotel, maintaining movement between the hotel and hospital while exclusively caring for this patient. No occupational exposures were reported during treatment. Following discharge, these healthcare workers underwent 21 consecutive days of health monitoring. All infection control measures were implemented under on-site guidance and verification by WCHSCU IPC specialists.

### Management of contacts

#### Management of close contacts

In this epidemiological investigation, six close contacts were identified within the hospital. Among them, close contacts 1, 4, 5, and 6 may have directly interacted with the patient’s blood, body fluids, or secretions while providing care. Close contacts 2 and 3 were potentially exposed to the patient’s blood or body fluids during sample processing (Table 3). These six individuals were placed in isolation for 21 days at the same hotel, with full salary maintained by the hospital throughout the isolation period to ensure compliance. Infection control professionals were responsible for planning their entry routes into the hotel.

**Table 3:** Information on close contacts

No.	Personnel type	Exposure circumstances
1	Resident physician in training	Nucleic acid samples were collected from the case without wearing gloves
2	Laboratory Technologist	Detection of absolute T-lymphocyte count, without wearing a mask
3	Laboratory Technologist	Blood sample testing conducted without wearing a mask
4	Professional caregivers	Performed urine disposal, oral care, and bed unit organization for the patient case without wearing gloves
5	Professional caregivers	Performed urine disposal and turning of the patient for the case without wearing gloves
6	Professional caregivers	Performed daily living care, and urine disposal for the case without wearing gloves

During the isolation period, none of the close contacts were permitted to leave their rooms, and all necessities were delivered to their rooms. They were required to monitor their symptoms and temperature twice daily, reporting the results to the infection control professionals. If any close contact’s temperature rose to ≥ 37.3 °C or if they developed symptoms suggestive of Lassa fever, medical personnel from the fever clinic conducted on-site specimen collection. To enhance early detection sensitivity, we defined fever as ≥ 37.3 °C based on the lower diagnostic threshold in diagnostics textbook [11]. On the 7th day and the 21st day after their last contact with the patient, nucleic acid testing for viral RNA was performed on sputum, urine, and blood specimens from all close contact. Throughout the health monitoring period, none of the close contacts exhibited abnormal temperatures or symptoms. Therefore, all six individuals were released from isolation after completing the 21-day quarantine.

#### Management of general contacts

In this epidemiological investigation, a total of 74 general contacts were identified within the hospital, including 21 doctors, 29 nurses, 8 technicians, and 16 logistics staff. General contacts were required to monitor their symptoms and temperature twice a day and report the results to the infection control professionals. If their temperature rose to ≥ 37.3 °C or if they developed symptoms suggestive of Lassa fever, they were instructed to immediately visit the fever clinic for testing.

During the health monitoring period, two general contacts developed acute fever. Laboratory testing revealed that one was infected with rhinovirus, and the other with the novel coronavirus. Samples were also sent to the provincial CDC for Lassa fever nucleic acid testing, with all results coming back negative.

## Discussion

This case of Lassa fever did not result in hospital-acquired infections. This can primarily be attributed to hospital staff’s adherence to standard precautions, with only 3.75% (3/80) of staff failing to choose PPE in accordance with established prevention protocols. The hospital mandates that all staff receive training on standard precautions upon joining the clinical department. Each ward is required to be equipped with a PPE box, and all staff must be aware of its location and proficient in the proper selection and use of PPE. Infection control professionals and link-nurses conduct periodic on-site guidance in wards to ensure staff are properly following standard preventive measures. Healthcare workers must rigorously implement Standard Precautions and correctly utilize PPE during routine clinical practice, irrespective of a patient’s provisional diagnosis. As demonstrated in the 2018 Nigerian outbreak where four healthcare workers died after exposure to an undiagnosed surgical patient with hemorrhagic symptoms [12]. In epidemiological investigations of Lassa fever hospital-acquired infections in Africa, the lack of PPE availability and training has been considered as a key factor contributing to outbreaks. This may also explain why our hospital’s management of the Lassa fever case did not result in an infection [12]. Another critical factor in preventing the spread of infection was the rapid initiation of the epidemiological investigation. Based on our experience in responding to public health emergencies, the hospital established an in-house emergency epidemiology team since 2020. This team comprises infection control professionals, nurses, and administrative personnel from various departments. They have all received epidemiological investigation training, and the hospital has developed a standard operating procedure (SOP) for such investigations. Normally, these team members perform their daily duties, but when an epidemiological investigation is required, they are mobilized from their posts to form the investigation team. Third, upon receiving a report of a Lassa fever case, we immediately contact the CDC, who assist our epidemiological team on-site with screening of suspected individuals and other tasks. This collaboration enhances the accuracy of our investigation results and speeds up the identification of potential cases. Fourth, During the epidemiological investigation, we adopted a multidisciplinary approach. In addition to the Infection Control Department, which was responsible for the investigation and response, the Medical Affairs Department organized multidisciplinary consultations for the case, the Nursing Department oversaw the patient’s care and sampling, clinical departments managed the patient’s transfer and isolation, the Equipment and Supplies Department ensured the availability of medical supplies, and the Infrastructure and Operations Department handled cleaning and disinfection of the ward.

We also conducted a review of the weaknesses in our hospital’s infection control measures during the management of this case. First, our ability to promptly detect special infectious diseases needs to be strengthened. Although this patient underwent pre-screening and triage upon admission, we failed to suspect Lassa fever based on the initial test results and clinical findings. Due to the nonspecific early symptoms of Lassa fever, early detection and differential diagnosis of the disease have always been key challenges in infection control [13–15]. In some healthcare facilities within endemic regions, broader infectious disease surveillance measures have been implemented. Early symptoms of Lassa fever, such as fever, headache, sore throat, cough, nausea, vomiting, diarrhea, muscle pain, chest pain, hearing loss, and contact with rodent excreta or known Lassa fever patients, employ in the definition of suspected cases [14, 16]. However, in non-endemic regions, where the probability of Lassa fever is extremely low, expanding the symptom surveillance criteria may not be efficient. We propose that infectious disease surveillance in non-endemic areas utilize information systems for non-specific monitoring, which could help in the early detection of infectious diseases while maintaining efficient screening. This model has been preliminarily applied and validated in Sierra Leone [17]. Medical institutions can utilize information systems to gather information on patients’ epidemiological history, symptoms, diagnoses, and test results, which can signal potential infectious diseases. Mathematical models can then be used for early warning, enabling non-specific identification of suspected cases. If there is a change in the types of infectious diseases prevalent locally, healthcare institutions can adjust their early warning criteria dynamically. For Lassa fever, for example, research has shown that certain symptom combinations are effective in predicting early cases, such as the combination of fever and gastrointestinal symptoms (aOR = 2.15; 95% CI 1.50–3.10) or fever and neurological symptoms (aOR = 6.37; 95% CI 1.49–27.16), further confirming the feasibility of using information systems to identify suspected Lassa fever cases [18]. Suspected cases should have clear isolation criteria, including those where malaria treatments are ineffective, symptoms persist after three days of malaria treatment, or individuals returning from Lassa fever-endemic regions present with symptoms of viral hemorrhagic fever. These cases should be isolated with the same precautions as confirmed Lassa fever cases before diagnosis [13, 14, 16, 19].

In this epidemiological investigation, two laboratory staff members were classified as close contacts due to not wearing masks while handling specimens from a Lassa fever case. Laboratory personnel are at risk of exposure to the blood, body fluids, and secretions of Lassa fever patients, which could lead to infection, especially before the case is confirmed. In one instance, a laboratory-acquired case of Lassa fever was reported in the United States, an investigator contracted the virus during manipulation of infected murine tissue cultures [20]. Therefore, during an epidemiological investigation, laboratory staff who handled specimens from Lassa fever patients should also be considered as potential exposed individuals, in addition to those who had direct contact with the patient. According to WHO guidelines, BSL-4 containment is required for procedures involving live Lassa virus culture or concentrated infectious materials, inactivated samples can be tested on lower containment facility [21]. For many healthcare institutions do not have such high-level laboratories, some studies suggest that specimens from suspected Lassa fever cases could be inactivated through chemical methods, gas fumigation, or heat treatment [20, 22]. Until such confirmation is obtained, sending specimens to specialized NGS laboratories represents a more feasible approach for achieving rapid and definitive identification of the pathogenic microorganism. Healthcare institutions should establish criteria for identifying suspected Lassa fever cases and provide staff training on these protocols. If a patient meets the criteria for suspected Lassa fever, specimens must be inactivated prior to shipment for NGS testing. This is one of the measures currently adopted by our hospital to ensure early detection of Lassa fever cases.

## Electronic supplementary material

Below is the link to the electronic supplementary material.

Supplementary Material 1

## Data Availability

No datasets were generated or analysed during the current study.

## Abbreviations

WCHSCU: West China hospital of Sichuan university
IPC: Infection prevention and control
NGS: Next generation sequencing
CDC: Center for disease control and prevention
PHCC: Public health clinical center of Chengdu
PPE: Personal protective equipment
ATP: Adenosine triphosphate
SOP: Standard operating procedure

## References

[1] Lassa fever n.d. https://www.who.int/news-room/fact-sheets/detail/lassa-fever (accessed September 27, 2024).

[2] Asogun DA, Günther S, Akpede GO, Ihekweazu C, Zumla A. Lassa fever: epidemiology, clinical features, diagnosis, management and prevention. Infect Dis Clin North Am. 2019;33:933–51. 31668199 10.1016/j.idc.2019.08.002

[3] Garnett LE, Strong JE. Lassa fever: with 50 years of study, hundreds of thousands of patients and an extremely high disease burden, what have we learned? Curr Opin Virol. 2019;37:123–31. 31479990 10.1016/j.coviro.2019.07.009

[4] Simonsen L, Kane A, Lloyd J, Zaffran M, Kane M. Unsafe injections in the developing world and transmission of bloodborne pathogens: a review. Bull World Health Organ. 1999;77:789–800. 10593026 PMC2557743

[5] Fisher-Hoch SP, Tomori O, Nasidi A, Perez-Oronoz GI, Fakile Y, Hutwagner L, et al. Review of cases of nosocomial Lassa fever in nigeria: the high price of poor medical practice. BMJ. 1995;311:857–9. 7580496 10.1136/bmj.311.7009.857 PMC2550858

[6] Lassa fever - Nigeria. n.d. https://www.who.int/emergencies/disease-outbreak-news/item/2023-DON463 (accessed August 29, 2024).

[7] Ijarotimi IT, Ilesanmi OS, Aderinwale A, Abiodun-Adewusi O, Okon I-M. Knowledge of Lassa fever and use of infection prevention and control facilities among health care workers during Lassa fever outbreak in Ondo state, Nigeria. Pan Afr Med J. 2018;30:56. 30197747 10.11604/pamj.2018.30.56.13125 PMC6125309

[8] Linwen G, Wenzhi H, Jingwen L, et al. Practical research on precision epidemiological investigation on COVID – 19 epidemic situation in medical institutions. Chin J Infect Control. 2023;22:586–90.

[9] Kitching A, Addiman S, Cathcart S, Bishop L, Krahé D, Nicholas M, et al. A fatal case of Lassa fever in london, January 2009. Eurosurveillance. 2009;14:19117. 19215723

[10] Grahn A, Bråve A, Lagging M, Dotevall L, Ekqvist D, Hammarström H, et al. Imported case of Lassa fever in Sweden with encephalopathy and sensorineural hearing deficit. Open Forum Infect Dis. 2016;3:ofw198. 27975074 10.1093/ofid/ofw198 PMC5152670

[11] Wan XH, editor. Diagnostics. 9th ed. People’s Medical Publishing House; 2018.

[12] Dan-Nwafor CC, Ipadeola O, Smout E, Ilori E, Adeyemo A, Umeokonkwo C, et al. A cluster of nosocomial Lassa fever cases in a tertiary health facility in nigeria: description and lessons learned, 2018. Int J Infect Dis. 2019;83:88–94. 30930184 10.1016/j.ijid.2019.03.030

[13] Woyessa AB, Maximore L, Keller D, Dogba J, Pajibo M, Johnson K, et al. Lesson learned from the investigation and response of Lassa fever outbreak, margibi county, liberia, 2018: case report. BMC Infect Dis. 2019;19:610. 31296177 10.1186/s12879-019-4257-z PMC6624965

[14] Rohan H. Beyond Lassa fever: systemic and structural barriers to disease detection and response in Sierra Leone. PLoS Negl Trop Dis. 2022;16:e0010423. 35587495 10.1371/journal.pntd.0010423 PMC9159599

[15] Njuguna C, Vandi M, Liyosi E, Githuku J, Squire JS, Njeru I, et al. After action review of the response to an outbreak of Lassa fever in Sierra leone, 2019: best practices and lessons learnt. PLoS Negl Trop Dis. 2022;16:e0010755. 36197925 10.1371/journal.pntd.0010755 PMC9534430

[16] Hamblion EL, Raftery P, Wendland A, Dweh E, Williams GS, George RNC, et al. The challenges of detecting and responding to a Lassa fever outbreak in an Ebola-affected setting. Int J Infect Dis. 2018;66:65–73. 29138016 10.1016/j.ijid.2017.11.007

[17] Magoba B, Gebru GN, Odongo GS, Hedberg C, Elduma AH, Kanu JS et al. Digitalizing disease surveillance: experience from Sierra Leone. Health Policy Plan. 2025;40:85–96. 10.1093/heapol/czae039 PMC11724635 38813658

[18] Ochu CL, Ntoimo L, Onoh I, Okonofua F, Meremikwu M, Mba S, et al. Predictors of Lassa fever diagnosis in suspected cases reporting to health facilities in Nigeria. Sci Rep. 2023;13:6545. 37085507 10.1038/s41598-023-33187-y PMC10121657

[19] Choi MJ, Worku S, Knust B, Vang A, Lynfield R, Mount MR, et al. A case of Lassa fever diagnosed at a community Hospital-Minnesota 2014. Open Forum Infect Dis. 2018;5:ofy131. 30035149 10.1093/ofid/ofy131 PMC6049013

[20] Blacksell SD, Dhawan S, Kusumoto M, Le KK, Summermatter K, O’Keefe J, et al. The biosafety research road map: the search for evidence to support practices in the Laboratory-Crimean congo haemorrhagic fever virus and Lassa virus. Appl Biosaf. 2023;28:216–29. 38090357 10.1089/apb.2022.0044 PMC10712363

[21] World Health Organization. laboratory biosafety manual, 4th edition. Geneva: World Health Organization; 2020.

[22] Raabe V, Koehler J. Laboratory diagnosis of Lassa fever. J Clin Microbiol. 2017;55:1629–37. 28404674 10.1128/JCM.00170-17 PMC5442519

