# Supplementary material for: Infection prevention and control measures for emerging infectious disease: lessons learned from the first case of imported Lassa fever in China
Source: Antimicrob Resist Infect Control. 2025 Jul 1;14:74. doi: 10.1186/s13756-025-01597-4 (PMC12210627; doi:10.1186/s13756-025-01597-4)
Supplement: Supplementary file 1 — Supplementary Material 1 [file 13756_2025_1597_MOESM1_ESM.docx]

Supplementary

Questionnaire that was used to investigate

Patient Information

Name (Medical Record), female, 49 years old.

Treatment Timeline:

August 2, ~14:00: Admitted to Bed 4 in Emergency Resuscitation Bay 2.

August 2, ~17:00 to August 3: Transferred to Bed 154 in Emergency Resuscitation Bay 3.

Diagnostic Procedures:

August 2: Visited CT room for chest CT scan.

August 3:

Visited CT room for cranial CT scan.

Underwent lumbar puncture in the Emergency Debridement Room.

1. Your Name *

_________________________________

2. Gender *

| ○Male |
| --- |
| ○Female |

3. Department/Unit Affiliation: *

_________________________________

4. Tel *

_________________________________

5. Your Occupation Category*

| ○Attending Physician |
| --- |
| ○Registered Nurse |
| ○Visiting Physician |
| ○Visiting Nurse |
| ○Resident Physician (Standardized Training) |
| ○Resident Nurse (Standardized Training) |
| ○Intern Physician |
| ○Intern Nurse |
| ○Housekeeping Staff |
| ○Support Staff / General Worker |
| ○Security Staff |
| ○Professional Patient Sitter |
| ○Central Transport Staff |
| ○Other (Please specify) _________________ |

6. Employee ID / Badge Number  *

_________________________________

7. Did you have contact with the patient? *

| ○Yes |
| --- |
| ○No |

8. If YES, describe the nature of contact and any procedures performed with the patient.

_________________________________

9. If YES, what Personal Protective Equipment (PPE) were you wearing during contact with the patient.

_________________________________

10. ID Card Number (Complete ONLY if in contact)

_________________________________

11. Home Address (Complete ONLY if in contact)

_________________________________
